# Prevalence and Correlates of Zinc Deficiency Among Vietnamese Women of Reproductive Age in Ho Chi Minh City: A Single Hospital-Based Survey

**DOI:** 10.3389/fgwh.2021.733191

**Published:** 2021-12-03

**Authors:** Vinh Quang Nguyen, Phong Van Lam, Aya Goto, Tu Van T. Nguyen, Thanh Nhan T. Vuong, Tien Minh Nguyen, Minh Ha Nguyen, Anh Tuyet T. Truong, Truc Phuong T. Tran, Chien Duc Vo

**Affiliations:** ^1^Department of Obstetrics and Gynecology, Nguyen Tri Phuong Hospital, Ho Chi Minh City, Vietnam; ^2^Department of Nutrition and Dietetics, Nguyen Tri Phuong Hospital, Ho Chi Minh City, Vietnam; ^3^Center for Integrated Science and Humanities, Fukushima Medical University, Fukushima, Japan; ^4^Department of Obstetrics and Gynecology, Tan Tao University, Ho Chi Minh City, Vietnam; ^5^Department of Laboratory, Nguyen Tri Phuong Hospital, Ho Chi Minh City, Vietnam; ^6^Department of Internal Medicine, Nguyen Tri Phuong Hospital, Ho Chi Minh City, Vietnam

**Keywords:** zinc deficiency, Vietnam, reproductive health, clinical laboratory techniques, health education

## Abstract

**Background and Objectives:** Zinc is a micronutrient that plays an important role in metabolism, cell growth regulation, and differentiation. Vietnam has many population groups living in poverty. The daily food of Vietnamese people is mainly rice, which contains very little zinc. This cross-sectional study was conducted to determine the prevalence of, and factors related to, zinc deficiency in women of reproductive age.

**Methods and Study Design:** The sample population was non-pregnant women of reproductive age (18–49 years old) who visited Nguyen Tri Phuong Hospital Gynecological Outpatient Clinic. The subjects were interviewed and data on background characteristics, anthropometric measurements, and blood tests (serum zinc concentration, complete blood count, albumin, and ferritin) were collected.

**Results:** The prevalence of zinc deficiency, as defined by the International Zinc Nutrition Consultative Group (IZiNCG), was 85% [61/72; 95% confidence interval (CI) = 74–91%], and the prevalence of severe zinc deficiency was 37% (27/72; 95% CI = 26–50%). There were significant associations of albumin concentration, marital status, and past pregnancy history with severe zinc deficiency.

**Conclusions:** More than three-fourths of Vietnamese women of reproductive age had zinc deficiency at our study site in Ho Chi Minh City. This health issue requires greater attention in order to swiftly promote preventive actions, and further surveillance to confirm our study findings.

## Introduction

Zinc is a trace element that plays an essential role in metabolism, regulation of cell growth, and differentiation (1, 2). Zinc is essential for the functioning of over 200 metalloenzymes and plays an important role in a range of biochemical and immune functions (3–5). Zinc is also important in genetic material transcription and translation, and has specific structural functions for proteins and cell membranes (2). Therefore, zinc deficiency causes a variety of disorders, such as impairments in growth, mental health, and the immune system (2, 6, 7).

Recently, the importance of zinc in preventing the various clinical manifestations mentioned above, especially in low- and middle-income countries, has been gaining increased attention. In the human body, zinc is mainly bound to albumin (70%). The serum zinc concentration of healthy subjects is maintained within a narrow range of about 12–15 mmol/L (78–98 mg/dL), even in populations with significant zinc intake. However, unlike many other minerals, there is no zinc storage in humans, and intake from food is necessary. Globally, about 17% of the population is estimated to be at a risk of inadequate dietary zinc intake, and the number is as high as 22% in East and Southeast Asia (8). Moreover, in Vietnam, like many low-income communities in developing countries, many people are living in poverty, which is known as the main determinant of micronutrient intake (9). In addition, the staple of the Vietnamese diet is rice, which contains very little zinc but is high in phytic acid, a compound that tries to bind minerals like zinc to form phytate salts and inhibit absorption of zinc, as well as iron (1, 4, 9).

This compound has an especially strong effect on women of reproductive age, the embryo, and the fetus, due to their significant demand for functional minerals, and because zinc is an important nutrient in the normal development process of the human body, especially in the rapid development of organs and organ systems (2, 6). According to recent reviews, the prevalence of low zinc concentrations in Vietnamese women of reproductive age was reported to be as high as 67%, a level exceeded only in Africa (10–12). Our previous study among Vietnamese pregnant women found the prevalence to be 29% (13). Nutritional intervention is proven to increase zinc intake among Vietnamese women (14, 15), which indicates that the prevalence of zinc deficiency may be changing as the country undergoes rapid socioeconomic and lifestyle changes. Therefore, constant monitoring is needed to provide up-to-date zinc deficiency data. A positive example is a recent marked decline in the prevalence of anemia. According to national nutrition surveys, the anemia prevalence in women of reproductive age declined from 24% in 2000 to 12% in 2010 (12), while the proportion of people living with poverty also declined from 29% in 2002 to 14% in 2010 (16). This study was designed to determine the prevalence of, and factors related to, zinc deficiency in Vietnamese women of reproductive age living in metropolitan areas.

## Materials and Methods

This was a hospital-based, cross-sectional study conducted at the Gynecological Outpatient Clinic in Nguyen Tri Phuong Hospital from March 2018 to May 2019. It is a tertiary hospital with about 900 beds located in Ho Chi Minh City. The sample population was non-pregnant women of reproductive age (18–49 years old). The sampling procedure was convenience sampling from women visiting the outpatient clinic, whose chief complaints were mostly genital tract infections, leiomyomata, and pelvic pain, or a routine checkup. Exclusion criteria were having any acute diseases (infection, diarrhea) and/or chronic diseases (cardiovascular disease, kidney disease, diabetes, hypertension) that may affect serum zinc concentrations. All invited women were informed of the study and provided their written, informed consent. The sample size was estimated based on the reported prevalence of zinc deficiency in Asia, which ranged from 34 to 73% (1), with a margin of error of 7%.

Eligible women were weighed to the nearest 0.5 kg while wearing light clothes and without shoes or sandals using a Tanita scale. Their height was measured to the nearest 0.5 cm using a stadiometer. Their body mass index (BMI) was calculated, and classified as underweight at 18.5 kg/m^2^.

Participating women were asked about past pregnancies, demographics (age, marital status), socioeconomic status (education level, job, household ownership), and health behaviors (smoking, secondary smoking, milk, and vitamin intake). After answering all the questions, the blood pressure of each woman was measured by a midwife.

Subsequently, serum was collected using a stainless steel needle into a trace element-free red cap plastic serum tube without an anticoagulant. Serum zinc concentrations vary by time of day of the blood collection and fasting status of the individual, so the time of taking the blood sample and 8-h fasting status were noted to determine the zinc deficiency cutoff value. Blood samples were kept at 15–25°C until analyzed. Zinc deficiency was defined in accordance with the International Zinc Nutrition Consultative Group's (IZiNCG) recommendation (17) (based on NHANES II data) as under 10.7 μmol/L for women whose blood sample was taken in the morning and were fasting for more than 8 h, under 10.1 μmol/L for women whose blood sample was taken in the morning and who were not fasting, and under 9.02 μmol/L for women whose blood sample was taken in the afternoon. Severe zinc deficiency was defined using a cutoff value of 7.65 μmol/L, following Wessells et al.'s recommendation (18). Standardization and calibration were carried out regularly using Olympus 480 apparatus. Albumin, ferritin, and the complete blood count were also evaluated.

Data were analyzed by Minitab 17 software. Descriptive analyses of demographic, socioeconomic, and biochemical data are listed in Table 1. Univariate analyses were carried out to identify relationships between zinc deficiency (both degrees of severity) and socioeconomic and nutritional status and biochemical indices. Since BMI is known to be associated with serum zinc concentration, and is an indicator of nutritional status, as well as albumin concentration (19), bivariate analysis was also performed for the albumin concentration. Similarly, age is reported to associate with zinc concentration, as well as pregnancy history and marital status, and thus bivariate analyses were performed for these two items by entering age.

**Table 1:** Zinc deficiency and associated factors.

	***Mean (SD)*** **or N (%)**			
	**Total**	**Deficient**	**Normal**	***p* ^†^**
	**(*n* = 72)**	**(*n* = 61)**	**(*n* = 11)**	
**Basic characteristics**				
Age (y)	*36.9 (7.2)*	*36.6 (7.3)*	*39.0 (6.2)*	0.262
Pregnancy history (no)	14 (19.4%)	11 (18.0%)	3 (27.3%)	0.438
**Anthropometry**				
Low BMI (<18.5 kg/m^2^)	5 (6.9%)	3 (4.9%)	2 (18.2%)	0.164
**Socioeconomic items**				
Educational level (primary school or lower)	9 (12.5%)	8 (13.1%)	1 (9.1%)	1.000
Marital status (married)	61 (84.7%)	52 (85.3%)	9 (81.8%)	0.671
House ownership (yes)	39 (54.2%)	26 (42.6%)	7 (63.6%)	0.198
**Personal behavior**				
Secondary smoking (yes)	23 (31.9%)	21 (34.4%)	2 (18.2%)	0.484
Milk supplementation (yes)	13 (18.1%)	12 (19.7%)	1 (9.1%)	0.676
Vitamin supplementation (yes)	9 (12.5%)	8 (13.1%)	1 (9.1%)	1.000
**Blood tests**				
Albumin (g/L)	*42.9 (2.1)*	*42.8 (2.1)*	*43.8 (2.2)*	0.160
Hb (g/L)	*125.6 (12.2)*	*124.6 (12.0)*	*130.8 (12.3)*	0.119
Ferritin (μg/L)	*77.1 (76.3)*	*76.6 (78.2)*	*79.8 (67.5)*	0.890
Anemia (Hb <120 g/L)	21 (29.2%)	20 (32.8%)	1 (9.1%)	0.158
Low Ferritin (≤30 μg/L)	28 (38.9%)	23 (37.7%)	5 (45.5%)	0.740

† *Independent Chi-squared test, t-test, or Fisher's exact test*.

This study was reviewed and approved by the Scientific Research Committee of Nguyen Tri Phuong Hospital (Approval No. 14 on June 24, 2016). Written informed consent was obtained. In cases where a participant's zinc concentration was found to be low, a nutritionist would contact them and offer advice on a zinc-rich diet.

## Results

A total of 115 women were invited to participate in the study. None met the exclusion criteria of having acute infections or chronic diseases. Of the 115 invitees, 34 refused to participate, and 9 were missing lab test results, resulting in a total sample size of 72 participants. The mean age of the women was 37 years, and 19% had no history of pregnancy (Table 1). Overall, 7% of participants in this study had a BMI <18.5 kg/m2, which is considered underweight according to World Health Organization (WHO) criteria. As for socioeconomic factors, 13% of women had an educational level of primary school or lower, and 54% owned a house. None smoked, but 32% women were exposed to passive smoking. In addition, 18% had daily milk intake, and 13% took vitamin supplements.

**Figure 1:**
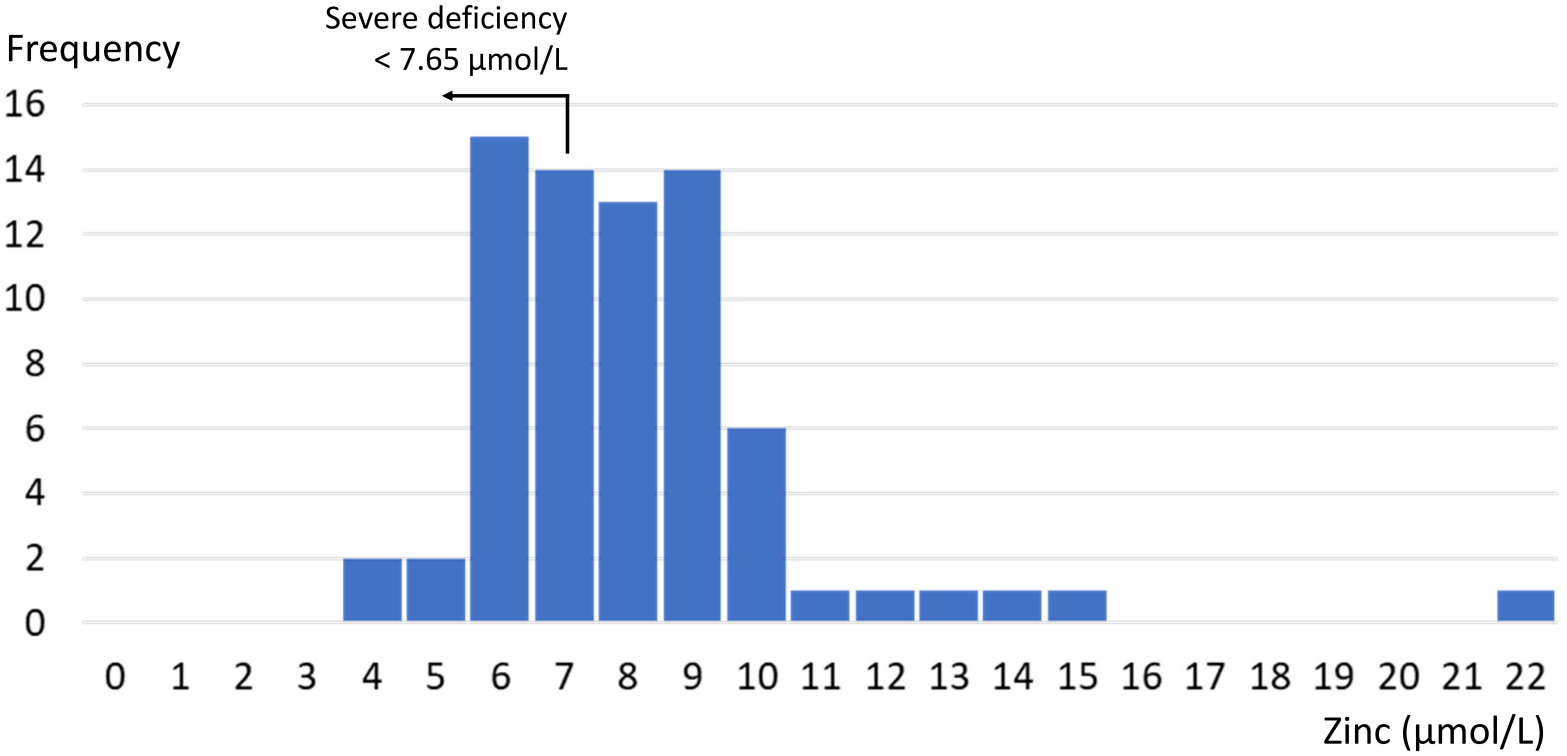
Distribution of serum zinc concentration.

The mean zinc concentration was 8.6 μmol/L [standard deviation (SD) = 2.6 μmol/L]. Figure 1 shows the distribution. The prevalence of zinc deficiency according to the IZiNCG definition was 84% (61/72; 95% CI = 76–93%). Mean albumin, ferritin, and hemoglobin levels (and SD, in brackets) were 42.9 (2.1) g/L, 77.1 (76.3) μg/L, and 125.6 (12.2) g/L, respectively; 29% of women had anemia according to the WHO criteria, and 39% had low ferritin levels (below 30 μg/L) (20).

As shown in Table 1, there were no significant associations between the zinc concentration and all tested variables. When focusing on the severe category, there were significant associations with past pregnancy history, marital status, and albumin concentration, as shown in Table 2. After controlling for BMI, albumin concentration remained significant (adjusted odds ratio = 0.72; 95% CI = 55–94%).

**Table 2:** Severe zinc deficiency and associated factors.

	***Mean (SD)*** **or N (%)**			**Univariate**	**Bivariate^‡^**
	**Severe deficiency**	**Other**	***p* ^†^**	**OR**	**OR**
	**(*n* = 27)**	**(*n* = 45)**		**(95% CI)**	**(95% CI)**
**Basic characteristics**					
Age (y)	*38.7 (6.0)*	*35.9 (7.7)*	0.082		
Pregnancy history (no)	2 (7.4%)	12 (26.7%)	0.046	0.86 (0.73–1.01)	0.88 (0.74–1.06)
**Anthropometry**					
Low BMI (<18.5 kg/m^2^)	1 (3.7%)	4 (8.9%)	0.644		
**Socioeconomic items**					
Educational level (primary school or lower)	3 (11.1%)	6 (13.3%)	1.000		
Marital status (married)	26 (96.3%)	35 (77.8%)	0.044	7.43 (0.89–61.72)	5.71 (0.65–50.53)
House ownership (yes)	18 (66.7%)	21 (46.7%)	0.099		
**Personal behavior**					
Secondary smoking (yes)	9 (33.3%)	14 (31.1%)	0.845		
Milk supplementation (yes)	6 (22.2%)	7 (15.6%)	0.535		
Vitamin supplementation (yes)	5 (18.5%)	4 (8.9%)	0.281		
**Blood test**					
Albumin (g/L)	*42.1 (1.9)*	*43.5 (2.1)*	0.004	0.72 (0.55–0.93)	0.72 (0.56–0.94)
Ferritin (μg/L)	*79.5 (96.3)*	*75.6 (62.4)*	0.851		
Anemia (Hb <120 g/L)	8 (29.6%)	13 (28.9%)	0.947		

† *Independent Chi-squared test, t-test, or Fisher's exact test*.

‡ *Adjusted with age for pregnancy history and marital status, and with BMI for albumin*.

## Discussion

In this single hospital-based study, the prevalence of zinc deficiency among Vietnamese women of reproductive age was 84%, similar to that of Nepal (78–90%) (21). This prevalence was higher than in previous studies in Vietnam (67%; 1,023/1,522) (10), as well as in Pakistan (42–48%) (11, 22) and Cambodia (45–63%) (11, 23). Although there is no gold standard method for determining zinc deficiency in the body, the WHO, UNICEF, IAEA, and IZiNCG recommend using serum zinc concentrations to assess zinc status (24). According to the IZiNCG's zinc risk assessment guide, zinc deficiency prevalence of above 20% in a population (or a population subgroup) alerts to the risk of zinc deficiency of the whole population and the need for intervention. Our results taken together with previous reports suggest that Vietnam is one such country.

There was a difference in mean albumin levels between the severe deficiency group and the other group. This significant association remained even when adjusted for BMI. This result may show that a low zinc concentration reflects a woman's nutritional status (25). Although this study was performed in Ho Chi Minh City, which is one of the largest economic centers in Vietnam, the prevalence of zinc deficiency was surprisingly high. Its association with albumin as an indicator of nutritional status was also noteworthy. However, there was no association with underweight or anemia, which would make clinical detection of zinc deficiency difficult. The serum zinc test is not covered by insurance, but it only costs 45,000 VND (about 2.00 USD), and we recommend its wider use to verify our findings at a larger scale.

There were significant associations between severe zinc deficiency and a past pregnancy history and being married in univariate analyses. However, it was not possible to confirm if these associations remained in a multivariable analysis model, mainly due to the small sample size. As mentioned above, further studies at multiple sites with a larger sample are necessary to identify high-risk groups.

The prevalence of anemia and iron deficiency in this study were 29 and 39%, respectively, which had no statistically significant association with zinc deficiency. This result suggests that a woman without anemia is still at risk of zinc deficiency. Although zinc-rich foods are often rich in iron, this study found no association between zinc and ferritin concentrations, which is a similar finding to that of a recent study of micronutrients in a Vietnamese population showing a gap between the prevalence of anemia and zinc deficiency (26). There may be other anti-nutrients in the Vietnamese diet that reduce zinc absorption, or additional iron may be provided through another source. Although the most recent Cochrane review states that the use of zinc-only supplementation in pregnancy remains controversial and that routine supplementation is not recommended (27), it advocates the use of multiple-micronutrient supplements with iron and folic acid in low- and middle-income settings, in line with previous Cochrane reviews (28). Such intervention is expected to improve low birthweight, small for gestational age, and possibly preterm births (28). Furthermore, maternal nutrition in the preconception period influences fetal growth and child development (29), and a recent study in Vietnam reported that preconception micronutrient supplementation had a positive impact on offspring's intellectual functioning at ages 6–7 years (30). If a high prevalence of zinc deficiency among Vietnamese women is confirmed through community-based studies, a more comprehensive approach toward improving multiple-micronutrient intake is recommended, rather than a single-nutrient approach.

This study has three major methodological weaknesses. First, data on detailed dietary habits (e.g., protein intake), other than milk and vitamin intake, were not collected. Second, BMI was the only indicator used to assess nutritional status. It is recommended that nutritional assessment criteria such as Nutrition Risk Screening-2002 or the Subjective Global Assessment be used. Third, as mentioned above, the sample size was not large enough, leading to the absence of a statistical model that could help explore complex associations of factors leading to zinc deficiency. Furthermore, our limited sampling at a single tertiary hospital in an urban area might have reflected the characteristics of patients visiting the hospital in terms of their demographic and socioeconomic backgrounds, resulting in limited generalizability of the results obtained. For example, the proportion of those with an education level of primary school or lower was much lower in our study participants of reproductive age (12.5%) compared with the number reported in a national nutrition survey (38%) (12). Despite these limitations, this study showed a notably high prevalence of zinc deficiency even among women visiting a tertiary hospital in a large city, who were assumed to have a relatively high socioeconomic status.

## Conclusion

This study showed that the prevalence of zinc deficiency in reproductive age women at our study site in Ho Chi Minh City was 85%. Zinc deficiency and the importance of a zinc-rich diet warrant greater attention from health professionals and the general public. Further studies with various population groups and detailed dietary information are needed to discuss specific ways to prevent and treat zinc deficiency among women in Vietnam.

## Data Availability Statement

The datasets presented in this article are not readily available because the IRB approval of the present study does not permit the data to be shared outside the hospital research team. Requests to access the datasets should be directed to Phong Van Lam, bsphongdinhduong@gmail.com.

## Ethics Statement

The studies involving human participants were reviewed and approved by Nguyen Tri Phuong Hospital. The patients/participants provided their written informed consent to participate in this study.

## Author Contributions

VN, PL, and TVN contributed to the study design, and all members to planning the data collection procedures. TV, TMN, AT, and TT collected data, MN was in charge of the quality control of laboratory tests, and CV supervised the whole process. VN and PL analyzed data, and all members participated in interpretation of obtained results. VN and PL wrote the first draft, and AG took part in revision of the paper. All authors contributed to the article and approved the submitted version.

## Funding

This project was supported in part by the Japan International Cooperation Agency Partnership Program Promoting Evidence-based Patient-centered Health Services in Southern Vietnam: University & Medical Association Partnership Initiative (PI: AG) and the Nguyen Tri Phuong Hospital research grant.

## Conflict of Interest

The authors declare that the research was conducted in the absence of any commercial or financial relationships that could be construed as a potential conflict of interest.

## Publisher's Note

All claims expressed in this article are solely those of the authors and do not necessarily represent those of their affiliated organizations, or those of the publisher, the editors and the reviewers. Any product that may be evaluated in this article, or claim that may be made by its manufacturer, is not guaranteed or endorsed by the publisher.
